# Non–Small Cell Lung Cancer Radiogenomics Map Identifies Relationships between Molecular and Imaging Phenotypes with Prognostic Implications

**DOI:** 10.1148/radiol.2017161845

**Published:** 2017-07-20

**Authors:** Mu Zhou, Ann Leung, Sebastian Echegaray, Andrew Gentles, Joseph B. Shrager, Kristin C. Jensen, Gerald J. Berry, Sylvia K. Plevritis, Daniel L. Rubin, Sandy Napel, Olivier Gevaert

**Affiliations:** From the Stanford Center for Biomedical Informatics Research, Department of Medicine (M.Z., O.G.), Department of Radiology (A.L., S.E., A.G., S.K.P., D.L.R., S.N.), Division of Thoracic Surgery, Department of Cardiothoracic Surgery (J.B.S.), and Department of Pathology (K.C.J., G.J.B.), Stanford University, 1265 Welch Rd, Stanford, CA 94305-5479.

## Abstract

**Purpose:**

To create a radiogenomic map linking computed tomographic (CT) image features and gene expression profiles generated by RNA sequencing for patients with non–small cell lung cancer (NSCLC).

**Materials and Methods:**

A cohort of 113 patients with NSCLC diagnosed between April 2008 and September 2014 who had preoperative CT data and tumor tissue available was studied. For each tumor, a thoracic radiologist recorded 87 semantic image features, selected to reflect radiologic characteristics of nodule shape, margin, texture, tumor environment, and overall lung characteristics. Next, total RNA was extracted from the tissue and analyzed with RNA sequencing technology. Ten highly coexpressed gene clusters, termed metagenes, were identified, validated in publicly available gene-expression cohorts, and correlated with prognosis. Next, a radiogenomics map was built that linked semantic image features to metagenes by using the *t* statistic and the Spearman correlation metric with multiple testing correction.

**Results:**

RNA sequencing analysis resulted in 10 metagenes that capture a variety of molecular pathways, including the epidermal growth factor (EGF) pathway. A radiogenomic map was created with 32 statistically significant correlations between semantic image features and metagenes. For example, nodule attenuation and margins are associated with the late cell-cycle genes, and a metagene that represents the EGF pathway was significantly correlated with the presence of ground-glass opacity and irregular nodules or nodules with poorly defined margins.

**Conclusion:**

Radiogenomic analysis of NSCLC showed multiple associations between semantic image features and metagenes that represented canonical molecular pathways, and it can result in noninvasive identification of molecular properties of NSCLC.

*Online supplemental material is available for this article.*

## Abstract (synopsis)

Our study presented a radiogenomics map of non–small cell lung cancer that linked image phenotypes with ribonucleic acid signatures captured by metagenes and showed their association with molecular pathways.

## Introduction

Non–small cell lung cancer (NSCLCnon–small cell lung cancer) is the most common type of lung cancer and is composed of tumors with significant molecular heterogeneity resulting from differences in intrinsic oncogenic signaling pathways (1). Molecular characteristics of NSCLCnon–small cell lung cancer formed the basis of clinical lesion diagnosis and therapeutic treatment (2,3). For example, the activation of the epidermal growth factor (EGFepidermal growth factor) receptor pathway determines treatment with anti-EGFepidermal growth factor receptor therapy by using tyrosine kinase inhibitors (4). Such molecular properties of NSCLCnon–small cell lung cancer were recently characterized (5) by quantitative imaging signatures. These findings and similar results in other cancers (6–8) confirm the potential synergy of integrating imaging and genomic data (9), yielding insights into understanding lesion-specific features and transcriptional regulators in patients with NSCLCnon–small cell lung cancer.

**Figure 1:**
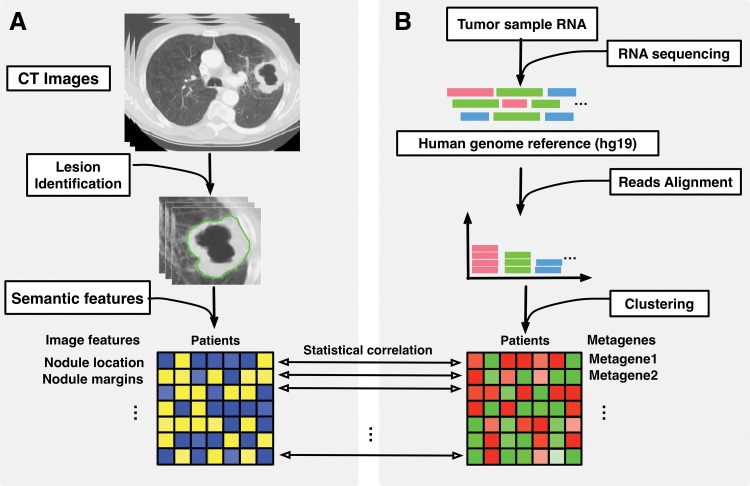
Overview of radiogenomic analysis to identify associations between, *A*, semantic features at CT and, *B*, RNA sequencing data.

Recent development of RNA sequencing techniques presents new opportunities for characterization of molecular pathways in NSCLCnon–small cell lung cancer (10,11). Accordingly, the emergence of radiogenomics (7,12,13) allows for identification of noninvasive biomarkers, which reflects cellular and molecular properties of NSCLCnon–small cell lung cancer. These noninvasive imaging biomarkers act as surrogates for molecularly defined features and may enable noninvasive precision medicine. Our radiogenomics analysis has several advantages over previous studies (Fig 1) (14,15). First, for the study of each patient, we collected an extensive collection of semantic features that consisted of 87 features defined by using a controlled vocabulary and that reflected radiologic characteristics of lung nodules (eg, nodule location, shape and texture of the tumor, and features derived from the lesion macroenvironment such as presence and patterns of emphysema and fibrosis). Second, we used RNA sequencing to characterize the transcriptomic profile of each tumor. We summarized these data by defining metagenes as clusters of coexpressed genes and used gene-enrichment analysis to annotate these metagenes with distinct molecular pathways. Additionally, we studied molecular prognostic significance by measuring overall survival predictors from public cohorts, which showed the prognostic associations of each metagene in two important NSCLCnon–small cell lung cancer histologic structures: adenocarcinoma and squamous cell carcinoma. The purpose of our study was to create a radiogenomic map that linked features from computed tomographic (CT) images and gene expression profiles generated by RNA sequencing for patients with NSCLCnon–small cell lung cancer.

## Materials and Methods

### Image Data Collection and Annotation

With institutional review board approval, we studied 113 patients who underwent surgery for NSCLCnon–small cell lung cancer between April 2008 and September 2014 at two medical centers and who underwent pretreatment chest CT examination and had tissue available for RNA sequencing (Table 1). Image data were obtained from GE medical systems (Waukesha, Wis) and Siemens (Erlangen, Germany) scanners. CT section thickness was as follows: less than 1 mm (22 patients, 19.5%), 1 mm to less than 2 mm (88 patients, 77.9%), and 2–3 mm (three patients, 2.6%). We developed a lung annotation template to facilitate selection of up to 87 semantic image features (Table E1 [online]) by using the open-source ePAD platform (*https://epad.stanford.edu*) that enables quantitative imaging annotations (16). These features reflected radiologic observations that included nodule shape, margin, texture, and location, and overall lung characteristics. A thoracic radiologist (A.L., with 20 years of chest oncologic imaging experience) annotated the CT image of each tumor by using ePAD while blinded to all clinical and molecular information. The semantic image features have binary values that reflect presence (or absence) of the radiologic features except for several variables that are ordinal in nature. These include nodule features that describe nodule margin (ie, smooth, irregular, lobulated, spiculated, and poorly defined), nodule shape (four classes from round to polygonal), nodule attenuation (four classes from pure solid to ground glass), and nodule ground-glass composition (6° from 0%–100%). We also evaluated for the presence, type, location, and distribution of emphysema and fibrosis. All features are reported in Table E1 (online). To remove features with less variant frequencies, we chose to study semantic features with occurrence rate of greater than 10% in the study cohort, which resulted in the removal of 52 features.

**Table 1:**
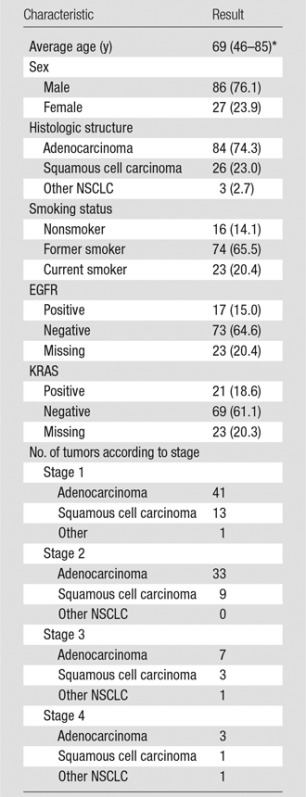
Clinical Characteristics of the Cohort

Note.—Unless otherwise indicated, data are number of patients and data in parentheses are percentages. EGFR = epidermal growth factor receptor, KRAS = V-Ki-ras2 Kirsten rat sarcoma viral oncogene homolog, Adeno = adenocarcinoma, SCC = squamous cell carcinoma.

*Data in parentheses are range.

### Next-generation RNA Sequencing Protocol

The tumor samples were frozen after surgery at −80°C until use. They were obtained intraoperatively as the specimen was removed from the patient. A slice of tumor cut from the center of the longest diameter was harvested (depending on the overall dimensions of the tumor, this slice thickness varied from several millimeters to nearly 1 cm). This process was completed within 30 minutes or less to minimize time before samples were frozen. For larger tumors we avoided any areas of obvious central necrosis. Tumor purity was verified for approximately one of every 10 lung tumors to be at least 50% surface area of a selected sample. Total RNA was extracted from nodule tissue samples and converted into a standard library of TruSeq Illumina kit for paired-end sequencing (Centrilion Biosciences, Palo Alto, Calif). The protocol included Ribo-Zero ribosomal RNA removal and ribosomal RNA depletion step, followed by fragmentation and complementary DNA with DNA synthesis by using SuperScript II (Life Technologies, Carlsbad, Calif). Quality was confirmed by using the BioAnalyzer (Agilent Technologies, Santa Clara, Calif), and the concentration was then evaluated by Kapa qPCR (Kapa Biosystems, Wilmington, Mass). RNA sequencing interpretations were mapped to the Human Genome version 19 (*http://genome.ucsc.edu*) by using the alignment algorithm (Star version 2.3; *https://github.com/alexdobin/STAR*). Next we used software (Cufflinks version 2.0.2; *http://cole-trapnell-lab.github.io/cufflinks/*) to determine the expression calls in each sample by using the number of fragments per kilobase of transcript per million mapped interpretations. Values of fragments per kilobase of transcript per million mapped interpretations were subsequently log transformed, and missing values were estimated by using a 15-nearest-neighbors algorithm. We only included transcripts with at least five interpretations mapped to their location in at least 70% of the samples.

### Statistical Analysis

By focusing on highly expressed gene expression data, we created metagenes with coherent gene expression as previously defined in Gevaert et al (15). We chose to select the top 10 metagenes with the highest cluster homogeneities in external gene expression data sets. We calculated the homogeneity score of each metagene by averaging all pairwise Pearson correlation coefficients of genes within the metagene and within each external gene expression data set. We used five public lung cancer gene-expression cohorts: the combined lung adenocarcinoma and squamous cell carcinoma from the Cancer Genome Atlas (17), the cohort from Lee et al (18), the cohort from Bild et al (19), the cohort from Shedden et al (20), and the cohort from Roepman et al (21), for a total of 1227 patients with NSCLCnon–small cell lung cancer. Next, we annotated the top 10 metagenes (Table 2) by using functional enrichment analysis of the metagenes (22). Briefly, we use the *P* value generated by a hypergeometric test that assessed whether the overlap between genes in a functional category and genes in a metagene was larger than expected by chance. We used the following databases that define functional categories for genes: MSigDB version 3 (23), GeneSetDB version 4 (24), Chea for Chip-X gene sets version 2 (25), and manually curated gene sets related to stem cells and immune gene sets. Gene set enrichment *P* values were corrected for multiple testing by using the false discovery rate (26), and we used a *P* value threshold less than .001 and *Q*-value threshold smaller than 0.05 to call a gene functional category significant.

**Table 2:**
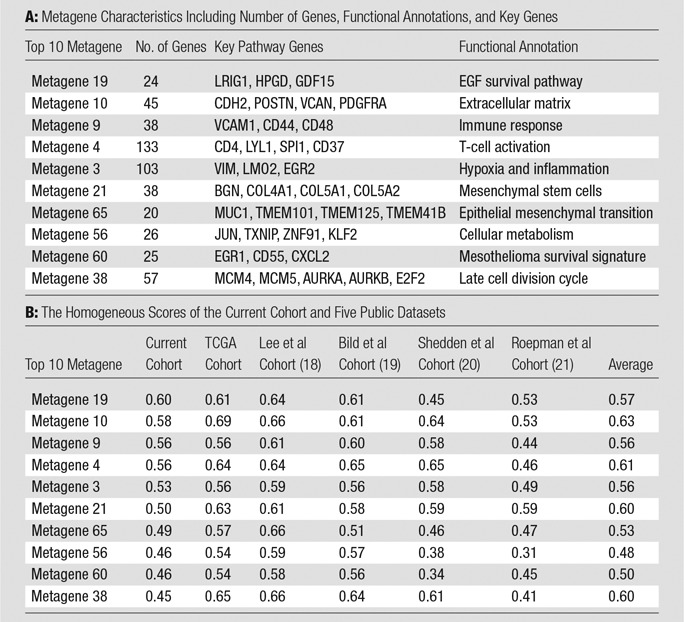
Metagene Characteristics and Homogeneity Scores

Note.—In part A, the homogeneous scores of the cohort and five public datasets were also recorded; metagenes were ranked in a decreasing order in terms of their homogeneity scores. In part B, metagenes were ranked in a decreasing order in terms of their homogeneity scores. The last column in part B shows the average records of the five validation cohorts. TCGA = the Cancer Genome Atlas.

We used PRECOG (*https://precog.stanford.edu/*) (27), a tool that links genes with prognosis by using the largest collection of gene expression data to date, to assess the survival relationship of the top 10 metagenes in adenocarcinoma and squamous cell carcinoma separately. PRECOG contains independent, publicly available gene-expression cohorts with survival data for lung adenocarcinoma (*n* = 17) and lung squamous cell carcinoma (*n* = 15). Within each metagene, the included genes were standardized to have no mean and unit variance in each cohort separately. For each dataset, association with overall survival was assessed by using univariate Cox proportional hazards regression (survival package version 2.38 in R version 3.1.2; R Foundation, Vienna, Austria). We reported the *z* score of each cohort to represent the direction and strength of the correlation of each metagene with overall survival (negative values indicated good prognosis and positive values indicated poor prognosis). Next, we computed a global meta-*z* score for each metagene by combining the *z* scores obtained from each public cohort by using the Stouffer method (27). A *z* score larger than 2 or smaller than −2 was considered to be statistically significant.

We built a radiogenomics map by associating metagenes with semantic image features. We used the *t* statistic and Spearman correlation metric to assess significant associations and used the false discovery rate (26) to correct for multiple testing. A *P* value less than .05 and a false discovery rate value less than 0.01 were used to determine statistically significant associations between metagenes and image features. We also reported image feature-to-metagene correlation coefficients to indicate directional relationship of metagenes and semantic features at CT imaging.

## Results

### Radiogenomics NSCLC Cohort

Table 1 shows the clinical characteristics of our cohort of 113 patients with NSCLCnon–small cell lung cancer. For each tumor, we used the previously described template (28) to annotate 87 semantic image features that represented the radiographic phenotype of each tumor (Table E1 [online]). Removal of low-variance features resulted in 35 semantic image features that captured nodule location, nodule margins, nodule attenuation, nodule ground-glass composition, and the presence of emphysema for subsequent statistical analysis. Next, we used high-throughput RNA sequencing to capture the transcriptome information of these cases. This RNA sequencing process resulted in quantification of 60 498 genes per sample represented by the ensemble identifiers, where transcripts that had at least five interpretations in 70% of the samples were included (see Materials and Methods section).

### Identification of Coexpressed Metagenes

We created the previously defined 56 metagenes in NSCLCnon–small cell lung cancer by using the RNA sequencing data collected here (9). Next, we validated the metagene homogeneity in five external public cohorts for validation (see Materials and Methods section ). Table 2b reports the metagene homogeneity in our cohort and the validation cohorts (metagenes were ranked in a decreasing order in terms of their homogeneity scores). A high value of homogeneity score indicates a high level of gene similarities within each metagene. We selected the top 10 metagenes with high average homogeneity (homogeneity score, ≥0.45) on the basis of their average score in validation cohorts for further analysis (Table 2b).

### Metagene Functional Enrichment and Prognostic Assessment

We annotated molecular functions of metagenes by using gene set enrichment analysis (23), which allows identification of shared common biologic pathways from a public molecular signature database. We demonstrated that the top 10 metagenes captured a variety of known molecular classes including EGF pathway (29), genes related to the extracellular matrix (30), immune response (31), and T-cell activation (32) (Table 2a). Next, we used survival meta-analysis (27) to assess the survival relationship of metagenes and public genomic cohorts with survival outcomes (Tables 3, E2 [online]). We showed that prognostic performance varied in groups of adenocarcinoma and squamous cell carcinoma. As shown in Table 3, five metagenes are significantly correlated with overall survival in adenocarcinoma data sets. For example, metagene 38 enriched by late cell division cycle genes is strongly correlated with poor prognosis, and four metagenes (metagenes 9, 19, 56, and 65) are correlated with positive survival. In squamous cell carcinomas, survival tended to be more heterogeneous with only three metagenes (metagenes 3, 4, and 9) and weakly correlated with good survival (Table 3).

**Table 3:**
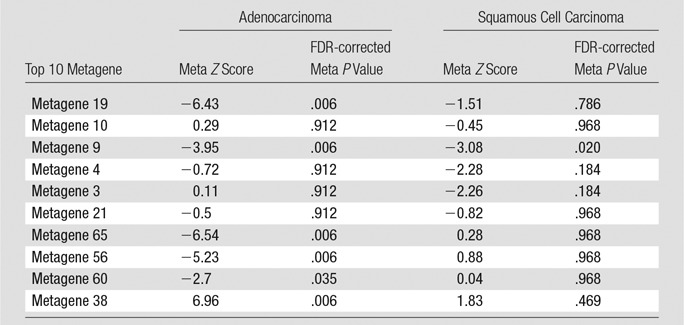
Prognostic Performance of Metagenes Assessed By Meta *Z* Scores Summarizing the Individual *z* Scores from PRECOG Analysis and Corresponding Meta *P* Value

Note.—Negative *z* scores indicate good prognosis and positive *z* scores indicate poor prognosis. See Table E2 (online) for detailed *z* scores for each individual adeno and squamous cell carcinoma data set. FDR = false discovery rate.

### Radiogenomics Map of NSCLC Revealed Associations

**Figure 2:**
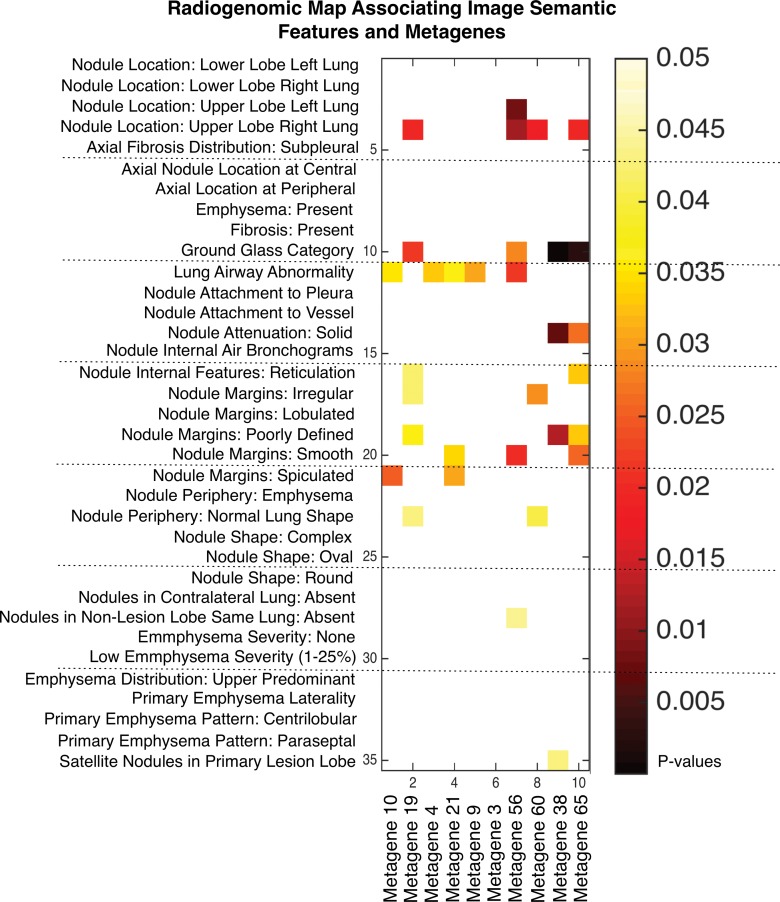
Radiogenomic map revealing 32 statistically significant associations between 35 CT semantic features and top 10 metagenes in NSCLCnon–small cell lung cancer. Only image features above the variance cutoff of 10% are shown. The order of image features and metagenes is determined by hierarchical clustering of the *P* values.

We built a radiogenomics map by correlating each image feature to the identified metagenes (Fig 2). We found 32 statistically significant correlations between semantic features and metagenes (Table 4; false discovery rate, <0.01). For example, we found that the metagene capturing the late cell cycle was associated with nodule attenuation and nodule margins. When this metagene was active, the lesion tended to be solid (metagene 38; false discovery rate, 0.01), whereas when this metagene was inactive, the lesion tended to have poorly defined margins (metagene 38; false discovery rate, 0.008). We also found that a normal lung background was positively correlated to a mesothelioma survival signature (metagene 60; false discovery rate, 0.008), which indicated that genes active in this pathway were associated with underlying lung parenchymal abnormalities such as emphysema when underexpressed and normal lung parenchymal morphologic structure when overexpressed. Next, metagene 65 was enriched with genes associated with the epithelial-to-mesenchymal transition and had several significant associations with image features (Table 4). For example, overexpression of metagene 65 was associated with poorly defined margins, and underexpression of metagene 65 was associated with smooth margins. Overall, our radiogenomic analysis captured multiple associations between semantic image features at CT and molecular pathway activity in NSCLCnon–small cell lung cancer (Fig 2, Table 4).

**Table 4:**
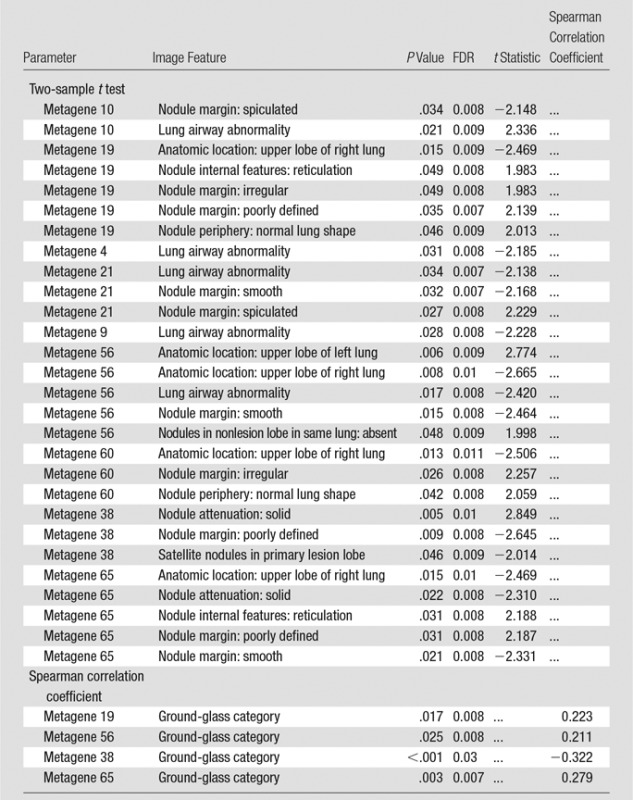
Associations between Image Features and Metagenes

Note.—The associations were ordered first by metagene and then by *P* value. *P* values and false discovery rate values presented statistical significance of the feature correlation. The *t* statistic is given for binary image features and the Spearmen correlation coefficient for ordinal image features. FDR = false discovery rate.

### EGF Pathway Characterizes Specific Images Phenotype of NSCLC

Next, we focused more deeply on the associations of metagene 19 with semantic features. Metagene 19 reflects the activity of the EGFepidermal growth factor pathway, which captures the relationship between the gene LRIG1 as a negative regulator of EGFepidermal growth factor. When metagene 19 is active, LRIG1 results in inhibition of cancer cell growth (33–35). Our radiogenomic map (Table 4) showed that metagene 19 was significantly associated with six semantic features at CT. Metagene 19 was negatively correlated with solid lesions and smooth margins (Fig 3a), which showed that low activity of metagene 19 was associated with solid lesions with smooth, lobulated, or spiculated margins. Conversely, high expression of metagene 19 was correlated with a high proportion of ground-glass opacity, irregular or poorly defined nodule margins, and reticulation (Fig 3b). More specifically, rising expression of metagene 19 was strongly correlated with nodule margin shape with increasing irregularity (Fig 4). Interestingly, this group of NSCLCnon–small cell lung cancer was enriched in cases with EGFepidermal growth factor receptor mutations (*P* < .0001, Wilcoxon rank-sum test).

**Figure 3a:**
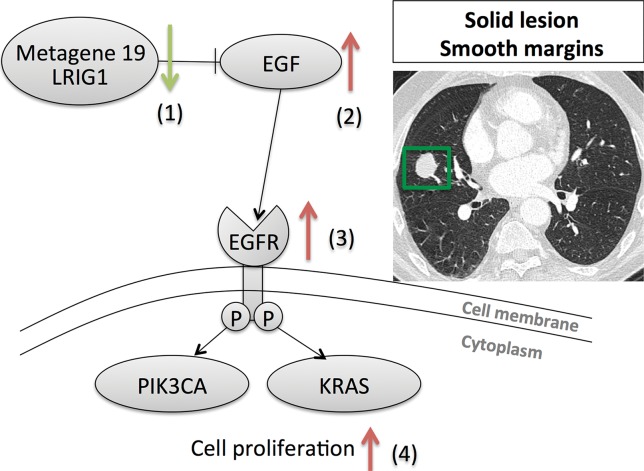
Association of the activity of metagene 19 with two distinct image phenotypes. **(a)** Low activity of metagene 19 is associated with low activity of LRIG1 (green arrow), and high activity of the EGF–EGF receptor (*EGFR*; red arrow) pathway results in cell proliferation through activating KRAS and PIK3CA. **(b)** High activity of metagene 19 results in high activity of LRIG1 (red arrow) with inhibition of the EGF–EGF receptor pathway (green arrow), but results in higher occurrence of EGF receptor mutations, which severs the link between LRIG1 and EGF receptor.

**Figure 3b:**
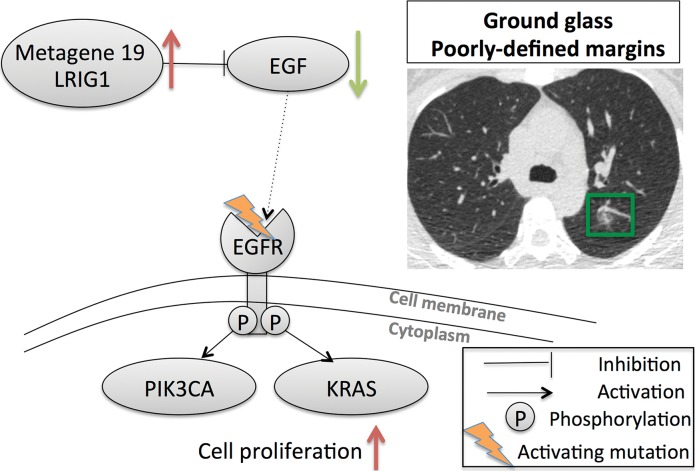
Association of the activity of metagene 19 with two distinct image phenotypes. **(a)** Low activity of metagene 19 is associated with low activity of LRIG1 (green arrow), and high activity of the EGF–EGF receptor (*EGFR*; red arrow) pathway results in cell proliferation through activating KRAS and PIK3CA. **(b)** High activity of metagene 19 results in high activity of LRIG1 (red arrow) with inhibition of the EGF–EGF receptor pathway (green arrow), but results in higher occurrence of EGF receptor mutations, which severs the link between LRIG1 and EGF receptor.

**Figure 4:**
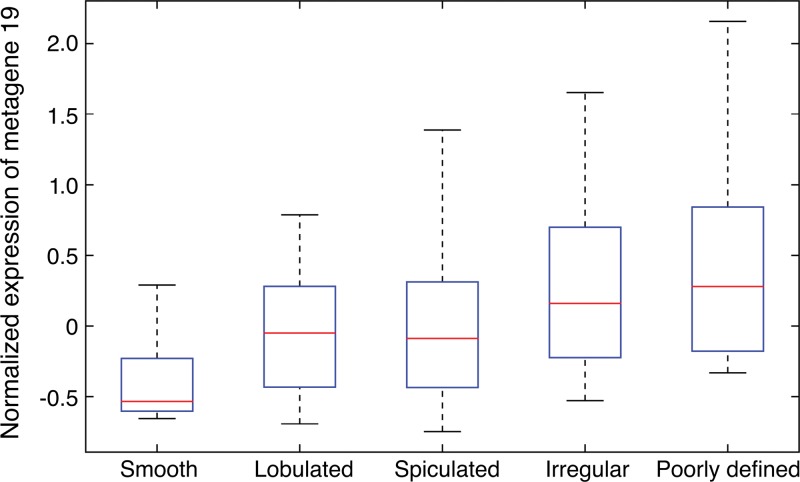
Box-and-whisker plot shows the distribution of the normalized expression of metagene 19 regarding different types of nodule margins at CT. The normalized expression of metagene 19 reflects the activity of the genes in this metagene.

## Discussion

In this study, we integrated semantic image features at CT with next-generation RNA sequencing data to identify radiogenomic biomarkers of NSCLCnon–small cell lung cancer. RNA sequencing analysis revealed 10 metagenes that resulted in 32 significant pair-wise associations between quantitative image features and metagenes annotated by functional gene enrichment analysis. These associations show the feasibility of noninvasive molecular characterization of NSCLCnon–small cell lung cancer by using radiogenomics mapping. We thoroughly validated the 10 metagenes in publicly available data sets by using their homogeneity, which reflected that coexpression is not only present in the cohort presented here, but also in five additional cohorts that represent 1227 patients from other institutes (Table 2). Moreover, correlation of the metagenes with clinical outcome in publicly available cohorts from patients from PRECOG (27) provided strong evidence of their prognostic significance in independently collected gene-expression cohorts (Table 3).

Linking imaging characteristics with molecular signatures is a growing field of research that provides additional value to clinical imaging with relevant molecular biology information (Table 4). For example, metagene 19 and its image-feature associations are a prototypical example of the possibilities of radiogenomics mapping (Fig 3). Low activity of this metagene reflects NSCLCnon–small cell lung cancer lesions that trigger the EGFepidermal growth factor receptor pathway, which consequently activates KRAS and PIK3CA genes and results in cell proliferation. We showed that these tumors are characterized by smooth margins and appear to be solid as defined by high attenuation at CT. However, high activity of metagene 19 corresponds to activation of LRIG1, a negative inhibitor of EGFepidermal growth factor that results in turning off the EGFepidermal growth factor receptor pathway. When these tumors are enriched in mutations in EGFepidermal growth factor receptor, thereby severing the inhibitory link with LRIG1, downstream activation of KRAS and PIK3CA pathways again occur but manifest with a different phenotype at CT as cancers with markedly irregular or poorly defined margins, most likely caused by the presence of ground-glass opacity. Overall, this example highlights that image phenotypes reflect different activities of molecular pathways and allow for noninvasive assessment of the molecular activity of NSCLCnon–small cell lung cancer lesions with potential implications for treatment. Moreover, we can speculate that the radiogenomic map can be extended to capture therapy response of existing or novel agents through the use of gene signatures predicting the response of treatment. These signatures can be mapped to image features by using radiogenomics mapping, as discussed here through metagenes, and subsequently allow for noninvasive assessment of treatment management.

Our study has the following limitations. Because the collected cohort of patients with NSCLCnon–small cell lung cancer included various section thicknesses and other acquisition parameters, future studies should determine the effects of scanner heterogeneity on the semantic annotations of radiologists. In addition, one thoracic radiologist annotated all semantic features for the study cohort. Future work that incorporates annotations by multiple radiologists is needed to study any potential variability in semantic feature annotation. Also, because this was a prospective cohort, direct survival analysis of the patients was not included because of lack of sufficient follow-up data of patients. To counter this, we introduced public datasets to associate metagenes with prognosis. We also opted to build a radiogenomics map in the largest possible cohort of NSCLCnon–small cell lung cancer. Therefore, we focused on all NSCLCnon–small cell lung cancers, including adenocarcinoma and squamous cell carcinoma. Although the histopathologic classification is readily distinguishable in tissue samples, it is not always apparent from the imaging phenotype.

In summary, in this study we presented a radiogenomics map of NSCLCnon–small cell lung cancer that linked image phenotypes with RNA signatures captured by metagenes and how they are associated with molecular pathways. This extensive radiogenomics map allowed for a better understanding of the pathophysiologic structure of lung cancer and how molecular processes manifest in a macromolecular way as captured by semantic image features. The presented image-to-molecular feature associations open possibilities for assessing therapeutic options on the basis of biologic pathway activity by using surrogate image features. Moreover, adding other molecular measurements such as DNA methylation or DNA copy number can deepen the radiogenomics associations and increase the potential of building radiogenomics maps even further, and enable a noninvasive in-depth understanding of lung cancer biology by using CT images.

**Advances in Knowledge**

■ Ten molecularly defined metagenes had 32 significant associations with CT image features in patients with non–small cell lung cancer (NSCLC) (false discovery rate, <0.01).
■ Radiologist-observed CT characteristics that captured nodule attenuation and nodule margins were associated with the late cell-cycle genes (false discovery rate, <0.01).
■ Radiologist-observed CT characteristics of the degree of ground-glass opacity and poorly defined and irregular margins of lung nodules were associated with the epidermal growth factor molecular pathway (false discovery rate, <0.01).

**Implication for Patient Care**

■ Multiple characteristics of lung nodules observed at CT by radiologists were associated with distinct molecular pathways in NSCLC, which may help guide clinicians in noninvasive treatment planning.

### SUPPLEMENTAL TABLES

Tables E1–E2 (PDF)
