# Supplementary material for: Non–Small Cell Lung Cancer Radiogenomics Map Identifies Relationships between Molecular and Imaging Phenotypes with Prognostic Implications
Source: Radiology. 2017 Jul 20;286(1):307–15. doi: 10.1148/radiol.2017161845 (PMC5749594; doi:10.1148/radiol.2017161845)
Supplement: Tables E1–E2 (PDF) [file ry161845suppa1.pdf]

©RSNA, 2017
10.1148/radiol.2017161845

Table E1. The CT Semantic Feature Template

Anatomic Location	1. Lower lobe of left lung
	2. Lower lobe of right lung
	3. Upper lobe of left lung
	4. Upper lobe of right lung
	5. Middle lobe of right lung
	6. Lingula
Axial Location	1 = Central | 2 = Peripheral (edge < 2 cm from visceral pleura)
Nodule Attenuation	1 = Solid
	2 = Pure Ground Glass (Nonsolid)
	3 = Part-solid, solidness > 5 mm
	4 = Part-solid, solidness < = 5 mm
Nodule Reticulation	1 = Absent | 2 = Present (lines inside ground glass nodule)
Internal Air Bronchograms	1 = Absent | 2 = Present
Nodule Necrosis	1 = Absent | 2 = Present
Nodule Cavitated	1 = Absent | 2 = Present
Nodule cysts	1 = Absent | 2 = Present
Primary Patterns of Nodule Margins	1 = Smooth (sharply delineated margins)
	2 = Irregular (minor oscillations or serrations of margin)
	3 = Lobulated (focal convexity or protrusions of lesion into lung)
	4 = Spiculated (linear radiations of finite length extending into adjacent lung)
	5 = Poorly defined (lack of clear delineation of margins)
Nodule Shape	1 = round (roughly spherical)
	2 = oval (ratio of x/y diameters > 1.5)
	3 = complex (neither 1 nor 2)
	4 = polygonal (straight or concave borders)
Nodule Calcification	1 = No calcification
	2 = Peripheral
Attachment to Pleura	1 = Absent | 2 = Present
Attachment to Vessel	1 = Absent | 2 = Present
Attachment to Bronchus	1 = Absent | 2 = Present
Pleural Retraction	1 = Absent | 2 = Present

Entering Airway	1 = Absent | 2 = Present
Thickened adjacent bronchovascular bundle	1 = Absent | 2 = Present
Vascular convergence	1 = Absent | 2 = Present
Septal thickening	1 = Absent | 2 = Present
Nodule Periphery	1 = Emphysema
	2 = Fibrosis (diffuse)
	3 = Normal lung shape
	4 = Scarring (focal)
Ground Glass Category	0 = 0% | 1 = 1%–25% | 2 = 26%–50% | 3 = 51%–75% | 4 = 76%–99% | 5 = 100%
Satellite nodules in Primary Lesion Lobe (≥ 4 mm, noncalcified)	1 = Absent | 2 = Solid | 3 = Part-solid
Nodules in Nonlesion lobe same Lung (≥ 4 mm, noncalcified)	1 = Absent | 2 = Solid | 3 = Part-solid
Nodules in Contralateral Lung (≥ 4 mm, noncalcified)	1 = Absent | 2 = Solid | 3 = Part-solid
Centrilobular Nodules–diffuse (RB type nodules)	1 = Absent | 2 = Present
Emphysema	1 = Absent | 2 = Present
Primary Emphysema Pattern	1 1 = Centrilobular
	2 2 = Panacinar
	3 3 = Paraseptal
Primary Emphysema Distribution	1 = Upper predominant
	2 = Middle Predominant
	3 = Lower Predominant
	4 = Diffuse, no predominance
	5 = Patchy, no predominance
Primary Emphysema Laterality	1 = Right | 2 = Left | 3 = Both
Overall Emphysema Severity	0 = None
	1 = Low (1%–25%)
	2 = Moderate (26%–50%)
	3 = Moderately High (51%–75%)
	4 = High (> 75%)
Airway Abnormalities	1 = Absent | 2 = Present
Bronchial Wall Thickening	1 = Absent | 2 = Present
Airway Ectasia (mild luminal enlargement)	1 = Absent | 2 = Present
Bronchiectasis (moderate enlargement)	1 = Absent | 2 = Present
Luminal Narrowing	1 = Absent | 2 = Present

Tree-in-Bud (airway secretions)	1 = Absent | 2 = Present
Fibrosis	1 = Absent | 2 = Present
Anatomic Fibrosis Distribution	1 = Upper predominant
	2 = Middle Predominant
	3 = Lower Predominant
	4 = Diffuse, no predominancne
	5 = Patchy, no predominance
Axial Fibrosis Location	1 = Subpleural
	2 = Bronchovascular
	3 = Both 1 & 2
	4 = Random
Fibrosis Type	1 = Usual Interstitial Pneumonia (UIP)
	2 = Nonspecific Interstitial Pneumonia (NSIP)
	3 = Smoking-related
	4 = Indeterminate
	5 = Others

Table E2. *Z* Scores of Each Metagene from Public Gene Expression Datasets That Were Collected from PRECOG

A: Adenocarcinoma Sets

GEO Number	GSE10245.ADENO	GSE1037.ADENO	GSE11117.ADENO	GSE13213.ADENO	GSE19188.ADENO	GSE29013.ADENO	GSE30219.ADENO	GSE31210.ADENO	GSE3141.ADENO	GSE31547.ADENO	GSE37745.ADENO	GSE42127.ADENO	GSE5843.ADENO	GSE8894.ADENO	GSE7878.ADENO	ca00182.ADENO	ca00191.ADENO
Metagene19	0.15	0.00	0.47	–3.20	–1.61	–1.66	–2.57	–2.80	–1.60	–1.72	–0.68	–3.21	–0.18	–1.05	–1.62	–2.97	–2.25
Metagene10	–0.59	–0.69	–1.32	–0.72	–0.21	0.86	2.05	0.77	0.97	0.73	–1.78	0.39	–0.84	–0.75	1.30	–0.56	1.59
Metagene9	–1.54	0.11	–0.85	–1.57	0.65	1.33	0.06	–1.63	–1.07	–0.97	–2.86	–1.20	0.39	–1.34	–1.22	–3.08	–1.49
Metagene4	–1.41	–0.32	–1.41	–2.12	1.54	1.37	1.40	0.75	–0.56	0.60	–1.77	–0.16	–0.71	0.93	0.00	–0.95	–0.15
Metagene3	–1.07	–0.82	–1.10	–1.54	1.14	1.02	2.09	0.61	0.66	0.66	–1.13	–0.28	–0.64	0.92	–0.38	–0.24	0.56
Metagene21	–0.31	–0.73	–1.52	–1.45	0.35	–0.13	1.38	1.11	1.41	0.48	–1.95	–0.06	0.04	–1.10	1.16	–1.84	1.11
Metagene65	0.33	–0.88	–0.51	–2.58	–2.16	–1.98	–3.15	–1.89	–1.60	–1.43	–1.64	–2.99	–0.14	–0.80	–0.50	–3.39	–1.64
Metagene56	–0.42	–1.07	–0.89	–2.03	–0.43	–0.05	–0.20	–2.69	–1.36	–1.44	–1.38	–2.13	0.21	–2.35	–2.02	–2.94	–0.38

Metagene60	−0.88	−0.94	1.83	−3.24	−0.54	−1.66	−0.96	−1.62	−0.15	−0.99	0.67	−2.17	0.89	0.29	−0.27	0.12	−1.51

B: Squamous Cell Carcinoma Sets

GEO Number	GSE10245.SCC	GSE1037.SCC	GSE11117.SCC	GSE13213.SCC	GSE19188.SCC	GSE29013.SCC	GSE30219.SCC	GSE31210.SCC	GSE3141.SCC	GSE31547.SCC	GSE37745.SCC	GSE42127.SCC	GSE5843.SCC	GSE8894.SCC	GSE7878.SCC
Metagene19	−1.09	0.23	−0.29	−0.94	−2.39	−0.23	1.22	0.25	−0.96	−0.78	−0.30	−0.94	−1.29	1.08	0.56
Metagene10	−0.74	−0.71	0.28	0.03	−0.76	0.01	0.59	−0.09	0.08	−0.33	−0.89	0.40	1.49	0.35	−1.43
Metagene9	0.34	−0.88	−1.65	−1.13	−1.80	−2.04	0.78	−0.49	−0.63	−1.21	−1.82	1.01	1.11	−1.80	−1.70
Metagene4	−0.46	−0.62	−1.22	−0.88	−2.12	0.06	0.85	−1.35	−0.31	−1.11	−0.77	0.50	0.74	−1.03	−1.09
Metagene3	−0.79	−0.73	−0.61	−0.85	−1.80	0.31	0.97	−1.48	−0.04	−1.04	−0.84	−0.63	1.13	−0.72	−1.64
Metagene21	−0.95	−0.24	0.70	−0.23	−0.82	−0.59	0.64	−0.17	0.06	−0.88	−0.96	−0.14	1.16	0.32	−1.08
Metagene65	−0.06	0.87	−0.36	−1.63	−1.33	−1.11	1.31	1.88	−1.33	−0.12	0.36	0.28	0.02	1.52	0.78
Metagene56	−0.30	−0.40	−0.26	−0.42	−1.10	−0.22	1.42	0.04	−0.88	−0.16	−1.24	3.09	3.19	0.92	−0.28
Metagene60	−1.24	−0.18	0.56	−0.81	−1.78	−0.05	1.44	0.41	−0.35	0.04	0.25	0.44	0.33	0.87	0.22

Note.—These sets reflect the association separately for adenocarcinoma in part A with 17 sets and for squamous cell carcinoma in part B with 15 sets.
